# Jellyfish Support High Energy Intake of Leatherback Sea Turtles (*Dermochelys coriacea*): Video Evidence from Animal-Borne Cameras

**DOI:** 10.1371/journal.pone.0033259

**Published:** 2012-03-16

**Authors:** Susan G. Heaslip, Sara J. Iverson, W. Don Bowen, Michael C. James

**Affiliations:** 1 Department of Biology, Dalhousie University, Halifax, Nova Scotia, Canada; 2 Department of Fisheries and Oceans, Population Ecology Division, Bedford Institute of Oceanography, Dartmouth, Nova Scotia, Canada; Hawaii Pacific University, United States of America

## Abstract

The endangered leatherback turtle is a large, highly migratory marine predator that inexplicably relies upon a diet of low-energy gelatinous zooplankton. The location of these prey may be predictable at large oceanographic scales, given that leatherback turtles perform long distance migrations (1000s of km) from nesting beaches to high latitude foraging grounds. However, little is known about the profitability of this migration and foraging strategy. We used GPS location data and video from animal-borne cameras to examine how prey characteristics (i.e., prey size, prey type, prey encounter rate) correlate with the daytime foraging behavior of leatherbacks (*n* = 19) in shelf waters off Cape Breton Island, NS, Canada, during August and September. Video was recorded continuously, averaged 1:53 h per turtle (range 0:08–3:38 h), and documented a total of 601 prey captures. Lion's mane jellyfish (*Cyanea capillata*) was the dominant prey (83–100%), but moon jellyfish (*Aurelia aurita*) were also consumed. Turtles approached and attacked most jellyfish within the camera's field of view and appeared to consume prey completely. There was no significant relationship between encounter rate and dive duration (*p* = 0.74, linear mixed-effects models). Handling time increased with prey size regardless of prey species (*p* = 0.0001). Estimates of energy intake averaged 66,018 kJ•d^−1^ but were as high as 167,797 kJ•d^−1^ corresponding to turtles consuming an average of 330 kg wet mass•d^−1^ (up to 840 kg•d^−1^) or approximately 261 (up to 664) jellyfish•d^-1^. Assuming our turtles averaged 455 kg body mass, they consumed an average of 73% of their body mass•d^−1^ equating to an average energy intake of 3–7 times their daily metabolic requirements, depending on estimates used. This study provides evidence that feeding tactics used by leatherbacks in Atlantic Canadian waters are highly profitable and our results are consistent with estimates of mass gain prior to southward migration.

## Introduction

Identifying the spatial and temporal characteristics of foraging habitat, search tactics, and diet of predators is fundamental to understanding their role in ecosystems and to developing conservation measures for threatened species, such as the protection of critical habitat. We expect animals to balance the benefits and costs of foraging decisions, since time and energy are spent searching for, capturing, and handling prey [1]. To begin to understand the foraging decisions of marine predators, it is important to study how prey characteristics (e.g., size of prey and patch density) influence their foraging behavior and success (e.g., [2], [3], [4]). However, prey are usually encountered and consumed at depth by marine animals, therefore, foraging behavior and diets are typically inferred indirectly, for instance from analyses of dive behavior and various diet estimate methods. The ability to directly observe and quantify foraging success in conjunction with understanding spatial movements over fine (1–10 km), meso (10s–100s of km), and large oceanographic scales (>1,000 km) is of great importance to better understanding marine animal populations and their variability [5].

The leatherback turtle (*Dermochelys coriacea*) is the largest living species of marine turtle, and also has the widest global distribution of any reptile. This species is listed as critically endangered globally [6] and endangered in Canada [7]. Leatherbacks undertake long-distance migrations (up to 18,000 km round-trip) between tropical breeding and foraging grounds and northern temperate foraging grounds [8], [9], [10]. Although east-west migrations are typical of some leatherback populations [8], [11] and return trips to specific foraging areas may span as long as 2–3 years, most sub-adult and adult leatherbacks in the northwest Atlantic perform these migrations annually [9], [12] to feed on gelatinous zooplankton, primarily jellyfish [13], [14], which are often associated with oceanographic features such as areas of upwelling [8]. For sexually mature adult leatherbacks, such migrations to high latitudes are presumably driven by the need to accumulate resources for reproduction [12]. However, during these migrations, leatherback turtles are exposed to a number of threats including fisheries bycatch (e.g., pelagic longline and particularly fixed gear in temperate waters [7]). In addition to human impacts, climate and oceanographic variability (which also influence prey distributions) no doubt also impact the life history of turtles in the Northwest Atlantic and are expected to influence juvenile recruitment and breeding remigration and contribute to range expansion (e.g., [5], [15]). Thus, it is of great importance to better understand leatherback foraging strategies to assess their significance to leatherback population energetics and to inform management measures such as the identification of critical habitat.

One of the most intriguing aspects of the foraging strategy of leatherback turtles is the almost complete reliance of such a large-bodied animal (up to 640 kg [12]) on a diet of gelatinous zooplankton, a low-energy food source [16], [17]. It has been estimated that hatchling leatherbacks may consume more than 100% body weight • day^-1^ [18] and adults at least 50% body weight • day^-1^ [19]. However, both the remote location of foraging and the sub-surface consumption of prey have precluded verification of such estimates. Found throughout the world's oceans, jellyfish are patchily distributed, but occur predictably at high densities in specific areas and at certain times of year [20]. Temperate coastal shelf waters of the North Atlantic are characterized by high concentrations of jellyfish during the summer months [21], [22]. Although dedicated studies of jellyfish distribution and abundance in Atlantic Canadian waters are lacking, spatial distributions of lion's mane jellyfish (*Cyanea capillata*), the largest extant species of jellyfish and a known prey of the leatherback turtle [14], are known to overlap with the occurrence of leatherback turtles (e.g., [23]).

Despite this overlap, the marine environment is dynamic, with prey often distributed heterogeneously within the landscape over space and time. The location of these prey are likely predictable at a large oceanographic scale, given the long-distance migrations of leatherback turtles and inter-annual fidelity to foraging areas [24], [25]. However, locating prey patches of jellyfish at meso-scales may be more difficult, as they vary spatially and temporally with influences from the movement of surface water and associated nutrients caused by wind [26] and tidal cycles. Because of this heterogeneity in prey presence with space and time, collecting simultaneous information about a predator's prey field and their movements [24], [25], [27] is necessary to try to understand an animal's foraging behavior. However, such sampling is expensive and logistically difficult.

Tracking data from satellite tags deployed over several months and over subsequent years have been used to explore the migratory movements of leatherback turtles, with foraging behavior inferred from diving behavior and dive-shape [9], [10], [28], [29], [30]. Such tracking data have been used to estimate behavioral states of leatherbacks based on changes in movement parameters such as speed and turning angle [31], [32], [33]. Movement data, along with concurrently collected dive data, have been used as a proxy for studying leatherback foraging (e.g., [34]), and sensors that can detect mouth opening [35], [36] and stomach temperature [37] may help determine the timing of prey capture events. Despite the utility of such methods, they are indirect measures of foraging since prey consumption is not observed.

The use of underwater animal-borne video cameras, in conjunction with electronic tagging technologies, provides the opportunity to directly observe foraging behavior. Such camera systems have been deployed on a variety of large marine predators, including pinnipeds, whales, sharks, and cheloniid turtles [38], [39], [40], [41]. Given the challenges associated with conducting in-situ studies of leatherback turtles at sea and recovering data loggers from free-swimming turtles, deployments of animal-borne cameras have been limited to nesting females and have not documented foraging [42]. However, the predictable occurrence of leatherback turtles off the coast of Canada during the summer months [43] provides the opportunity to study foraging leatherbacks when they are presumably acquiring the energy required for southward migration and, for many, reproduction. We attached an animal-borne video camera with an incorporated global positioning system (GPS) to free-ranging leatherback turtles in shelf waters off Nova Scotia, Canada. Our objectives were to describe prey-specific components of foraging behavior (e.g., encounter rate, capture success rate, and handling time), and to estimate daily energy intake, with the aim to better understand the profitability of migratory patterns and implications for characterizing critical foraging habitat of leatherback populations.

## Methods

### Ethics statement

This research was conducted in accordance with guidelines of the Canadian Council on Animal Care. The protocol was approved by the University Committee on Laboratory Animals, Dalhousie University's animal ethics committee (protocol numbers 08-077 and 09-069) and Fisheries and Oceans Canada (license and permit numbers 2007-024, MAR-SA-2007-006, 2008-454, MAR-SA-2008-006, 323395, 323398, and 326240). Instruments were attached to the carapace of free-swimming turtles without capture from a boat to reduce handling effects on the animals. During tracking, a minimum observation distance of ∼400 m was maintained to minimize the disturbance of turtles.

### Study area

The study was conducted in the temperate shelf waters off Cape Breton Island, Nova Scotia, Canada (approximately 47° N, 60°W). Instruments were deployed at a median distance of 13.1 km off the coast (*x*− = 15.7 km, range 3.1–35.0 km) during August and September 2007–2010. Previous studies have characterized aspects of this ecosystem and shown that a relatively large and predictable assemblage of sub-adult and adult leatherbacks feed in this area every year [9], [33], [43].

### Instruments and deployments

**Figure 1:**
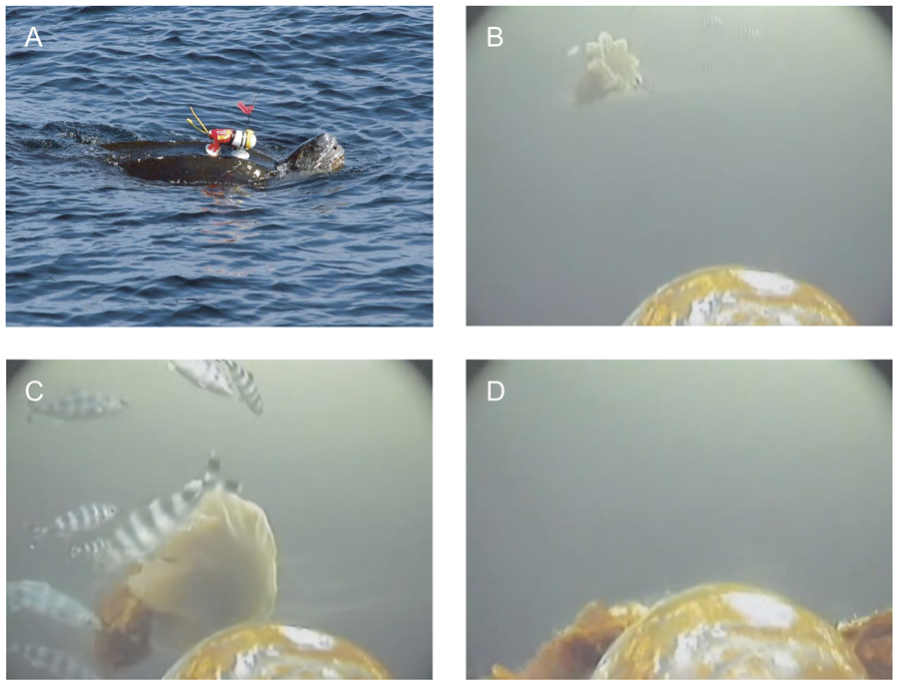
Serrano-V camera and example still images. Camera with suction-cup attachment to the shell of a leatherback turtle (a) and still images extracted from a video file recorded on 3 September 2010 showing a turtle approaching a lion's mane jellyfish that is surrounded by pilot fish, *Naucrates ductor*, (b, c) and subsequently consuming this jellyfish (d).

The Serrano-V (Fig. 1a, Xeos Technologies Inc., Bedford, NS, Canada) is a charge-coupled device color, video camera system (235×83 mm, 270 mm with antennae, 1013 g) which operates under low light, without the need for accessory lighting, and records 320×240 QVGA. The unit contains an integrated time-depth recorder unit (TDR; that also measures temperature), GPS receiver, suction cup attachment, remote release, and a 900 MHz spread spectrum two-way radio transceiver to command the unit. The video camera recorded continuously, and was turned on either prior to deployment, or remotely, after the camera was attached to the turtle. The video camera remained on the turtle until it either detached on its own, or was released remotely (≤4 hours). All camera deployments occurred during daylight hours to ensure that there was sufficient ambient light to quantify the components of foraging and to recover the instrument.

Leatherbacks basking and/or handling prey at the surface were approached by a 10 m commercial fishing vessel equipped with a 3 m bowsprit. Tags were hand-placed on the carapace just behind the head (Fig. 1a) from a rigid platform suspended from the bowsprit, approximately 0.5 m above the water's surface. When possible, turtles were captured after instrument recovery using a breakaway dip-net (for details see [30]). Curved carapace length (CCL; ± 1 cm) and width (CCW; ± 1 cm), sex (judged by tail length), and, when feasible, body mass (± 0.5 kg), was recorded. Turtles were equipped with metal flipper tags and a microchip implant (right pectoral muscle) so that recaptured individuals could be identified. The maximum width of the dorsal surface of the head (which was normally within the camera's field of view, e.g., Fig. 1b) was only measured for two of the turtles deployed with video cameras in 2010. We used these values, in addition to those of 21 other separately captured adult turtles in 2010, to represent the mean head width for all video-sampled turtles (mean = 23 cm, range 20.3–25.3, *n* = 23) in order to estimate the size of captured prey (see Video analysis and Energetic intake sections).

### Video analysis

Behaviors were scored using the event-recording software JWatcher [44]. The following behaviors were recorded: (1) time at surface (interval between dives); (2) time below surface (dive duration/search time); (3) prey detection (change in head direction); (4) capture/first contact with jellyfish; (5) bites/head movements associated with consuming jellyfish; (6) pursuit – the interval between prey detection and capture; (7) handling time – the interval from the time of capture until the last bite (in view) or contact with a subsequent jellyfish; and (8) capture success. Dives were defined as a time of submersion greater than 30 s. Number of jellyfish attacked and encounter rate per unit time was calculated for each dive. Prey size was estimated by comparing the jellyfish contracted bell diameter relative to the width of the turtle's head. To do this, we froze the video immediately prior to prey capture (e.g., Fig. 1c). This relative measure of jellyfish size was then converted to an absolute estimate using an average head width of 23 cm (see Instruments and deployments section). To standardize our estimates, when possible, we measured jellyfish in the contraction stage of movement with the bell draped down, since the demarcation of the bell edge was more defined in the contracted state and was less likely to extend beyond the field of view just prior to capture (e.g. Fig. 1d). Additionally, when an estimate of jellyfish size could be made from the video, the bell was most often in a contracted state near the head. Thus, the bell diameters we measured represent a minimum size (i.e., contracted state) in comparison to bell diameters observed during expansion in the video or measured when removed from the water and placed on a flat board (the most frequent method used for measuring jellyfish). We used the contracted-bell measurements to investigate the relative influence of prey size on handling time. Nevertheless, we noted many instances in the video when the diameter of jellyfish with expanded bells, just prior to capture, exceeded the width of the turtle's head and the entire field of view (e.g., Fig. 1d), indicating that turtles were consuming jellyfish >23 cm and of sizes more consistent with previous measurements made for this species (e.g., [17]).

### Spatial movement

Surface positions of turtles from the Serrano-V's integrated GPS unit were used to determine the spatial extent of turtle movements during foraging. GPS locations were used to calculate the distance travelled from the deployment location. Total distance travelled was not calculated as GPS fixes were not reliably obtained for each surfacing between dives for all turtles; instead, a single displacement value for each turtle was calculated as the maximum distance from the deployment position.

### Statistical analyses

Linear mixed models were used to analyze the effect of prey size and prey species on handling time, as well as the effect of dive duration on encounter rate. Separate models were also fitted to explore whether the displacement distance during the period of video sampling was related to the number of prey encounters, i.e., whether the distance between the capture and camera release locations was inversely related to prey encounter rate. The intercept of these models was permitted to vary randomly across animals. A first order autoregressive correlation structure (corAR1) was used to account for serial correlation among repeated measurements. Analyses were performed using the ‘glmPQL’ function of the ‘nlme’ package [45] in R 2.8.1 [46]. Residual plots and partial residual plots were examined to assess model fit and the normality of residuals were assessed with a two-tailed Kolmogorov-Smirnov test. All data are expressed as mean ± standard deviation.

### Energy intake

To estimate energy intake during foraging, we assumed that turtles were foraging on lion's mane jellyfish during daylight hours only (at this time of year: ∼13.5 hrs), that turtles encountered jellyfish at the mean encounter rate per minute of each turtle, and that the average jellyfish consumed had energy contents comparable to those sampled by Doyle et al. [17]. The assumption of daylight-only feeding is supported by concurrent research using stomach temperature telemetry of leatherbacks in the same study area and during the same time of year which indicates that foraging occurs primarily, if not exclusively, during the daylight hours (J. Casey, unpublished data). This assumption of daylight-only feeding is further supported by archival tags data that demonstrate diving behavior is largely limited to the photic zone, with pronounced diurnal changes in dive depth (K. Hamelin, unpublished data).

Since energy density values for lion's mane jellyfish were not available in our study, we used the average size and energy values determined for lion's mane jellyfish by Doyle et al. [17] for energy intake calculations: mean bell diameter 30.3±6.6 cm (range 15–47 cm, *n* = 27), wet mass 1263.1±662.3 g, and gross energy density 0.2±0.04 kJ g WM^-1^. Doyle's measurements were taken from freshly stranded jellyfish specimens (either on the beach or in the water close to shore) collected in the North Atlantic (Layton Beach, County Meath, Ireland; 53.67°N, 6.23°W) between July–October 2004. These size and energy values were also similar to those measured previously for lion's mane jellyfish in the northwest Atlantic (Newfoundland, Canada [47]). Given that these collections were in similar northern temperate waters and during the same season as our study, and that our observations of expanded bell size of jellies consumed overlap with those sampled by Doyle et al. [17], we use Doyle's energy content values as an appropriate proxy for the jellyfish being consumed in our study.

## Results

Video from the Serrano-V camera was recovered from 19 turtles (Table S1) during 2008 (*n* = 8), 2009 (*n* = 4), and 2010 (*n* = 7). Video duration averaged 1 hour and 53 minutes per turtle (range 0:08–3:38 h). In 2006–2007, when cameras had been deployed on turtles that were first captured, no foraging behavior was recorded (MC James, pers. comm.). However, placement of the camera on free-swimming turtles without capture in 2008–2010 resulted in no observed behavioral effects and foraging behavior was recorded for all camera deployments, suggesting that there was minimal effect of the camera on foraging.

### Foraging behavior, prey encounters and spatial movement

Eighteen of the 19 turtles foraged mainly on lion's mane jellyfish (range = 83–100% for each turtle), although moon jellyfish (*Aurelia aurita*) were also consumed. One of the 19 turtles was anomalous in that it was observed scavenging, had a low prey encounter rate, and 2 of the 5 jellyfish consumed were moon jellyfish. Commensal pilot fish (*Naucrates ductor*) were identified from the video for 4/19 deployments for all years and were observed swimming in the vicinity of the turtle's head and/or near lion's mane jellyfish that were approached and consumed by turtles (e.g., Fig. 1b,c).

Jellyfish were consumed at depth in all years, but 2010 was notable in that consumption of dead lion's mane jellyfish floating at the surface was also observed (range = 0–12% for 2010 deployments). The dive durations (3.22±1.77 min; range 0.32–6.84 min) and surface intervals (2.44±1.80 min, range 0.004–11.26 min; Table S2) we measured were within the range of values for an additional leatherback turtle equipped with a satellite-linked TDR, but no camera, that used the study area during the months of August and September, 2008 (dive duration 4.64±2.20 min; surface interval 3.32±2.90 min; median dive depth 21.5 m, range 5.5–97.0 m; K. Hamelin, unpublished data). Foraging at depth was restricted to the photic zone, and although the camera routinely switched from color to black and white mode with decreasing light levels at greater depths, there was always sufficient ambient light to identify prey encounters. Prey were encountered in 77±22% (range 29–100%) of dives (Table S2). Jellyfish encounter rates varied among dives and among turtles, with encounters per minute of diving averaging 0.60±0.44 and encounters per minute of video sampling averaging 0.37±0.22 (Table S2). There was no significant relationship between encounter rate and dive duration (*p* = 0.74; full details of the regressions are provided in Table S3). A total of 601 jellyfish captures were recorded and capture success was 100% for all turtles. Turtles attacked an average of 83±16% jellyfish within the field of view. These predation rates are an underestimate because, for some deployments, the field of view of the camera only included a small part of the head, thus it is possible that additional jellyfish may have been consumed by the turtle outside the field of view of the camera.

The straight-line distance turtles traveled from the position of camera deployment to position of camera release ranged from 0.72–9.02 km and turtles generally traveled away from the deployment position (Figure S1). There was an inverse correlation between prey encounter rate and total distance travelled from the deployment location (i.e., prey encounter rates were relatively lower in turtles traveling further from the deployment location, *p*<0.05).

### Prey size, handling time and energy intake

**Figure 2:**
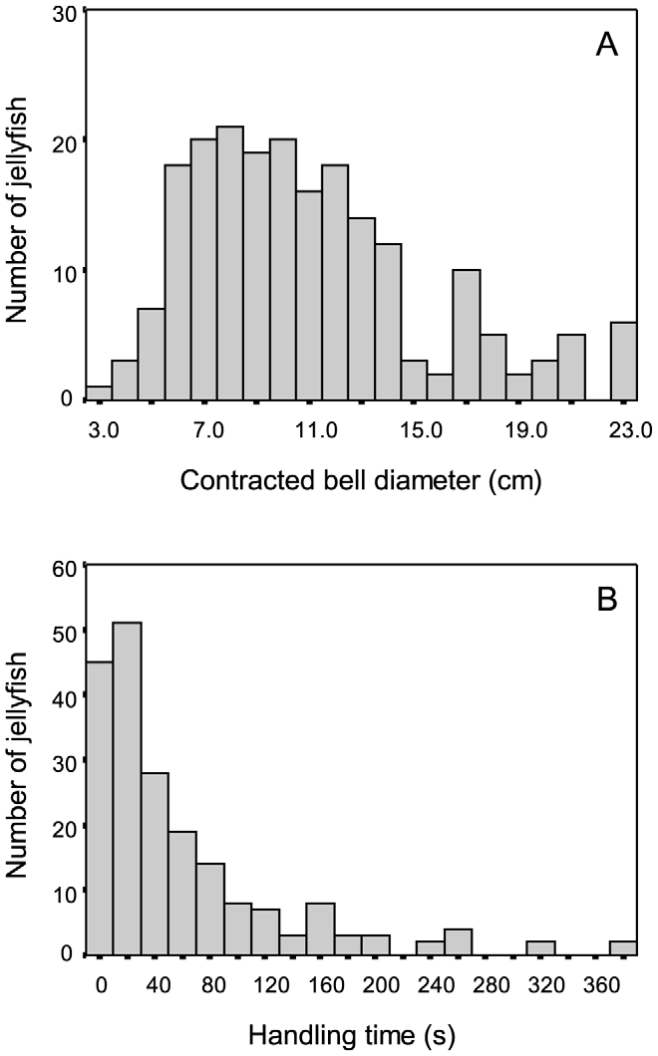
Frequency distribution of relative prey size (contracted bell diameter) (a) and handling time (b) of lion's mane jellyfish. Results represent the size distribution of measured lion's mane jellyfish (*n* = 350) captured by 19 leatherback turtles.

Six-hundred and ninety lion's mane jellyfish (593 captured) and 24 moon jellyfish (8 captured) were observed in the videos. Of the captured lion's mane jellyfish, 350 were measured to examine the relationship between relative prey size and handling time. Contracted bell diameter of lion's mane jellyfish consumed by turtles averaged 11.2±4.4 cm (range 3.1–22.7 cm; Fig. 2a) and moon jellyfish contracted diameter was estimated to be 4.6±2.1 cm (range 2.1–9.3 cm). These values underestimate jellyfish contracted size because it was not always possible to measure the contracted jellyfish right before capture (were measured at a greater distance from the camera), and the relative size of the turtle's head in the field of view differed somewhat among turtles due to the variable placement of the camera on the carapace. Pursuit time for lion's mane jellyfish was estimated to be 22.9±13 s (range 3–79 s) in two turtles for which the head was in view to observe a change in head direction that was assumed to correspond with prey detection. All prey attacked were mostly eaten, with no apparent preference for particular anatomy.

**Figure 3:**
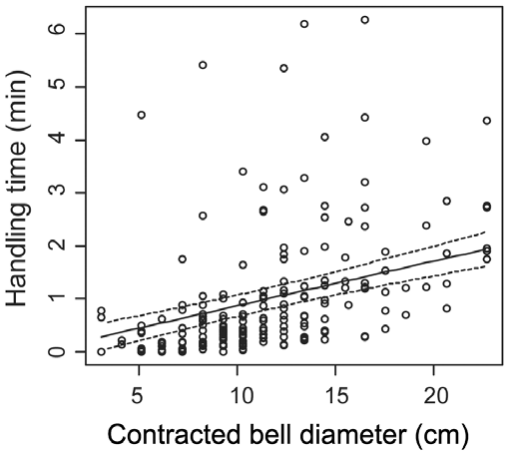
Effect of relative prey size (contracted bell diameter) on handling time of lion's mane jellyfish. Solid line represents mean predicted values with dashed lines indicating ± s.e.m. Circles represent observed values.

Handling time for lion's mane jellyfish was estimated to average 59.5±72 s overall, but ranged from 0 to 375 s (Fig. 2b). This wide range in handling time was explained by differences in prey size. That is, handling time increased significantly with increasing contracted bell diameters of the jellyfish consumed (*p* = 0.0001; Fig. 3 and Table S3), but there was no difference in the relationship between handling time and prey size between the two prey species (*p* = 0.92; Table S3). Following capture of prey at depth, turtles often continued processing jellyfish at the surface, which resulted in a relatively greater handling time for a similar prey size than may have been consumed during a dive. During a dive, handling time of jellyfish was often not complete before the subsequent jellyfish was encountered and attacked. Handling time of the last jellyfish encountered during a dive often had a relatively greater handling time than prey encountered earlier in the dive.

Individual estimates of energy intake averaged 66,018±42,034 kJ (range 315–167,797 kJ) per day, assuming a 13.5 hr period of daylight foraging (Table S2). These values represent a consumption of 330±210.1 kg (range 2–840 kg) wet mass per day or approximately 261 lion's mane jellyfish (range 1–664) per day.

## Discussion

The temperate waters off eastern Canada support one of the largest seasonal foraging populations of sub-adult and adult leatherback turtles in the Atlantic [34]. The 12,000–18,000 km round-trip migrations of leatherbacks from tropical and sub-tropical breeding areas to high latitude foraging areas in the western Atlantic is thought to have evolved to permit turtles to capitalize on seasonally-abundant prey in coastal temperate waters. James et al. [48] estimated an average ∼33% increase in mass of turtles before their initiation of southward migration. Although surface foraging by leatherback turtles has been opportunistically documented in this high latitude foraging area [14], until now, prey encounter rates, prey size, and handling times at depth had not been quantified nor had daily energy intake been estimated.

The range of prey encounter rates reported here presumably reflect patchily distributed jellyfish at fine spatial scales (100s of meters) [26], even though jellyfish were present in about 75% of dives. The high encounter rates of jellyfish per dive lend support to the identification of this area as a foraging “hotspot” for leatherbacks (e.g., [33], [48]). The importance of this foraging area is further supported by our estimate that turtles in this area consume an average of 66,018 kJ and up to 167,797 kJ per day. We were able to measure mass for only two of the turtles equipped with video cameras (mean = 455 kg; Table S1). However, mean curved carapace length of six of the turtles was 154 cm, which also roughly corresponds to a body mass of 455 kg [12], [48]. Thus, if we assume 455 kg was the average mass of individuals in our study, turtles consumed an average of 73% and up to 184% of their body mass per day in wet mass of jellyfish, equating to an average energy intake of 145 kJ•kg^−1^ or up to 369 kJ•kg^−1^ per day. The allometric relationship for the field metabolic rate (FMR, kJ•d^−1^) of an ectothermic reptile [49] suggests the predicted FMR for a 455 kg reptile would be 46.2 kJ•kg^−1^. Although it has been proposed that leatherback turtles demonstrate some metabolic endothermy (perhaps regionally), using doubly-labeled water, Bradshaw et al. [50] estimated the daily diving metabolic rate (DMR) of leatherbacks nesting in the tropics to be 20.7 kJ•kg^−1^ or less than half that predicted by for an ectothermic reptile of similar size (and an order of magnitude lower than a similarly-sized endotherm). These estimates were not dissimilar to earlier measurements of leatherback FMR made by Wallace et al. [51], supporting the conclusion that leatherbacks appear to be ectothermic and rely on large body size, insulating fat layers, and thermal inertia to regulate body temperatures above ambient [50]. The turtles in our study consumed an average of 3 (and up to 8) times their daily metabolic requirements as would be estimated by allometry, or 7 (up to 17) times their DMR as measured in nesting leatherbacks.

Although jellyfish are relatively energy-poor [17], our results demonstrate that leatherback predation on high densities of readily-captured lion's mane jellyfish results in high energy intake at least at this time of year, which is consistent with the estimated mass gain of leatherback turtles in Canadian waters. Jellyfish graze on copepods, larvaceans, cladocerans, and meroplankton [52], [53], and leatherbacks in turn graze on patches of these scyphomedusae which tend to ingest the relatively larger size component of available zooplankton prey [52], [54]. Although jellyfish are patchily distributed in time and space, oceanographic features and processes produce predictable foraging opportunities for leatherbacks such that the benefits of reliance on a diet of jellyfish apparently outweigh the energetic costs of migrating to these northern waters. Leatherbacks were not likely prey-limited in our study, as productivity of jellyfish in temperate coastal areas and particularly here in the strongest outflow of the Gulf of St Lawrence can yield excellent foraging opportunities. Also there seems to be little competition for jellyfish apart from niche overlap with ocean sunfish (*Mola mola*), a species which is also present in the study area during the same times of year.

Our data further suggest that leatherback turtles are efficient predators since no time was wasted on unsuccessful attacks, a foraging strategy similar to that of grazers. Also jellyfish appeared to be completely consumed. Predation rate on high density prey is likely to be limited by handling time or the animal becoming satiated [55]. We found some evidence for this prediction, as turtles while they were already handling other prey in ∼80% of those instances when jellyfish in the field of view of the camera were not targeted. The longer handling times of prey when turtles returned to the surface further suggests that turtles may require further handling for some jellyfish. Given the relatively simple body composition of jellyfish, it is unlikely that digestion time is limiting. Conversely, given the anatomy of lion's mane jellyfish, and particularly those with very large bell diameters and long tentacles, turtles likely face prey handling challenges. Therefore, it is understandable that even with the assistance of the leatherback turtle's specialized esophagus (with papillae pointing towards the stomach), consuming such prey may limit intake. We were unable to distinguish between handling and digesting prey, therefore, it is unclear how digestion may influence the foraging behavior of turtles.

Our estimates of leatherback turtle foraging behavior are based on relatively short-term video records compared to a leatherback's typical 3–5 month high-latitude foraging period in the Northwest Atlantic and, therefore, may not be representative of the entire period. Additional information on daily predation rates, sizes of prey consumed, and variability in energy contents of jellyfish in these northwest Atlantic waters during the summer and fall will be useful to refine these estimates. Nevertheless, our results offer evidence that the feeding tactics of leatherbacks in this high latitude coastal foraging area off Atlantic Canada are energetically profitable and are consistent with estimates of mass gain prior to southward migration and preparation for the breeding season. Longer deployments will be needed to confirm our estimates over time periods that have broader ecological implications, and to place the fine- to meso-scale foraging movements of leatherback turtles within the context of the large-scale migratory movements that have been previously described for this population. Further studies of the foraging decisions that turtles make would also benefit from the collection of concurrent conductivity-time-depth recordings, location and three-dimensional movement data, as well as better information on the prey field.

By simultaneously collecting video and high-resolution dive and ocean temperature data, the purpose-built camera we used to study leatherback foraging behavior during relatively short daytime periods may help confirm inferences of foraging from satellite tracking data that has been collected over much broader spatial and temporal scales. Therefore, this technology offers promise as a tool for determining critical areas of foraging habitat in support of conserving this endangered species.

## Supporting Information

Figure S1 **Distance between original camera deployment location and each surfacing location of 19 leatherback turtles as estimated from GPS locations.**
(TIF)

Table S1 **Instrument deployment details for 19 leatherback turtles.**
(DOC)

Table S2 **Dive and prey encounter data (mean±S.D.) for 19 leatherback turtles estimated from video, energy intake estimated from prey encounter rate, and speed and distance travelled estimated from GPS locations.**
^1^Estimated energy intake assuming encounter rate extrapolated over 13.5 hrs daylight and using average size and energy values for lion's mane jellyfish measured in Doyle et al. [17]. *Camera facing to the side or up, head not always in view. ^+^Dead jellyfish floating at the surface.
(DOC)

Table S3 **Parameter estimates and significance of model terms.**
This table shows the linear mixed model parameter estimates and significance of model terms for three models: the effect of jellyfish encounters per dive minute on dive duration, the effect of prey size on handling time, and the effect of prey size and prey species on handling time. The results show that jellyfish encounters per dive minute are positively correlated with dive duration, that prey size is positively correlated with handling time, and that the relationship between prey size and handling time does not differ among species. The hypothesis that the residuals of these fits follow a normal distribution is not rejected by two-tailed Kolmogorov-Smirnov tests (*p>*0.05).
(DOC)
