# Correction: Jellyfish Support High Energy Intake of Leatherback Sea Turtles (*Dermochelys coriacea*): Video Evidence from Animal-Borne Cameras

**DOI:** 10.1371/annotation/4722cac5-8305-4b03-b805-a59ced1eea49

**Published:** 2012-06-13

**Authors:** Susan G. Heaslip, Sara J. Iverson, W. Don Bowen, Michael C. James

There was an error in the corresponding author designation. Michael C. James is the only corresponding author. His email address is mcjames@dal.ca. 

