# Supplementary figures and images for: Jellyfish Support High Energy Intake of Leatherback Sea Turtles (Dermochelys coriacea): Video Evidence from Animal-Borne Cameras
Source: PLoS One. 2012 Mar 16;7(3):e33259. doi: 10.1371/journal.pone.0033259 (PMC3306388; doi:10.1371/journal.pone.0033259)

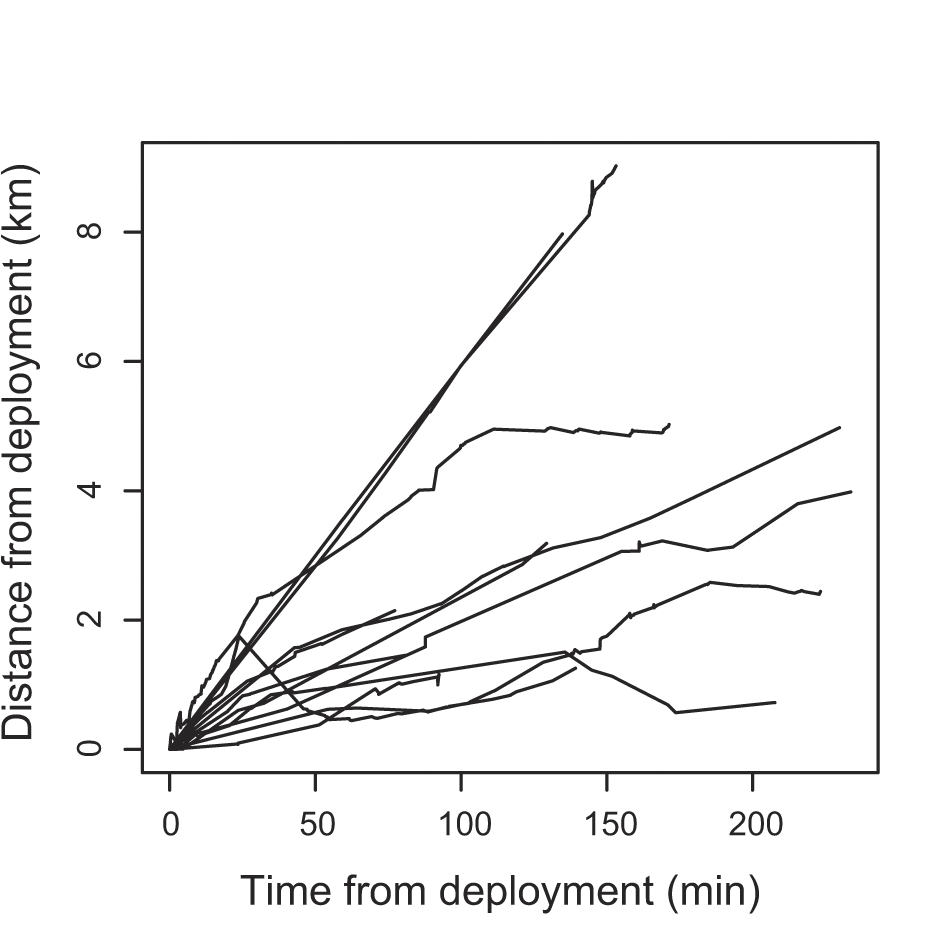

Supplement: Figure S1 — Distance between original camera deployment location and each surfacing location of 19 leatherback turtles as estimated from GPS locations. (TIF) [file pone.0033259.s001.tif]
