# Supplementary material for: Jellyfish Support High Energy Intake of Leatherback Sea Turtles (Dermochelys coriacea): Video Evidence from Animal-Borne Cameras
Source: PLoS One. 2012 Mar 16;7(3):e33259. doi: 10.1371/journal.pone.0033259 (PMC3306388; doi:10.1371/journal.pone.0033259)
Supplement: Table S1 — Instrument deployment details for 19 leatherback turtles. (DOC) [file pone.0033259.s002.doc]

| **Turtle ID** | **Date** | **Deployment time** | **Sex** | **Post-deployment capture** | **Curved carapace length (cm)** | **Curved carapace width (cm)** | **Weight (kg)** | **Head width (cm)** | **Video duration (h:m:s)** |
| --- | --- | --- | --- | --- | --- | --- | --- | --- | --- |
| A | 12 August 2008 | 9:24 | ♀ | Y | 153.1 | 114.5 | - | - | 1:02:50 |
| B | 12 August 2008 | 15:30 | ♂ | N | - | - | - | - | 2:15:49 |
| C | 13 August 2008 | 13:02 | - | N | - | - | - | - | 1:46:59 |
| D | 14 August 2008 | 9:44 | - | N | - | - | - | - | 0:07:59 |
| E | 14 August 2008 | 11:27 | ♀ | N | - | - | - | - | 3:08:58 |
| F | 16 August 2008 | 9:20 | - | N | - | - | - | - | 0:09:34 |
| G | 18 August 2008 | 14:02 | ♀ | N | - | - | - | - | 1:37:44 |
| H | 3 September 2008 | 10:33 | ♂ | Y | 150.7 | 108.3 | 460.0 | - | 1:12:39 |
| I | 29 August 2009 | 14:11 | ♀ | Y | 143.5 | 108.8 | 450.5 | - | 1:25:28 |
| J | 4 September 2009 | 11:29 | - | N | - | - | - | - | 0:44:59 |
| K | 8 September 2009 | 12:04 | ♂ | N | - | - | - | - | 1:56:48 |
| L | 12 September 2009 | 10:49 | ♀ | Y | 157.5 | 111.3 | - | - | 0:56:39 |
| M | 11 August 2010 | 11:51 | ♂ | Y | 158.2 | 120.6 | - | 25.2 | 2:38:18 |
| N | 14 August 2010 | 11:47 | ♀ | N | - | - | - | - | 2:17:18 |
| O | 15 August 2010 | 17:41 | ♀ | N | - | - | - | - | 2:15:46 |
| P | 16 August 2010 | 11:45 |  | N | - | - | - | - | 3:24:16 |
| Q | 24 August 2010 | 12:29 | ♂ | Y | 162.2 | 112.5 | - | 24.5 | 3:36:02 |
| R | 29 August 2010 | 10:12 | - | N | - | - | - | - | 3:38:52 |
| S | 3 September 2010 | 13:25 | ♂ | N | - | - | - | - | 1:43:23 |

|  |  |  |  |  |  |  |  |  |  |
| --- | --- | --- | --- | --- | --- | --- | --- | --- | --- |
|  |  |  |  |  |  |  |  |  |  |
|  |  |  |  |  |  |  |  |  |  |
|  |  |  |  |  |  |  |  |  |  |
|  |  |  |  |  |  |  |  |  |  |
|  |  |  |  |  |  |  |  |  |  |
|  |  |  |  |  |  |  |  |  |  |
|  |  |  |  |  |  |  |  |  |  |
|  |  |  |  |  |  |  |  |  |  |
|  |  |  |  |  |  |  |  |  |  |
|  |  |  |  |  |  |  |  |  |  |
|  |  |  |  |  |  |  |  |  |  |
|  |  |  |  |  |  |  |  |  |  |
|  |  |  |  |  |  |  |  |  |  |
|  |  |  |  |  |  |  |  |  |  |
|  |  |  |  |  |  |  |  |  |  |
|  |  |  |  |  |  |  |  |  |  |
|  |  |  |  |  |  |  |  |  |  |
|  |  |  |  |  |  |  |  |  |  |
|  |  |  |  |  |  |  |  |  |  |
