# Supplementary material for: Jellyfish Support High Energy Intake of Leatherback Sea Turtles (Dermochelys coriacea): Video Evidence from Animal-Borne Cameras
Source: PLoS One. 2012 Mar 16;7(3):e33259. doi: 10.1371/journal.pone.0033259 (PMC3306388; doi:10.1371/journal.pone.0033259)
Supplement: Table S2 — Dive and prey encounter data (mean±S.D.) for 19 leatherback turtles estimated from video, energy intake estimated from prey encounter rate, and speed and distance travelled estimated from GPS locations. 1Estimated energy intake assuming encounter rate extrapolated over 13.5 hrs daylight and using average size and energy values for lion's mane jellyfish measured in Doyle et al. [17]. *Camera facing to the side or up, head not always in view. +Dead jellyfish floating at the surface. (DOC) [file pone.0033259.s003.doc]

| **Turtle ID** | **Number of dives** | **Proportion of dives that prey were encountered** | **Dive duration (min)** | **Surface interval (min)** | **Jellyfish encounters (attacked (pass/unknown))** | **Jellyfish encounters per dive minute** | **Jellyfish encounters per minute** | **Energy intake^1^ (kJ/13.5 hr)** |
| --- | --- | --- | --- | --- | --- | --- | --- | --- |
| A | 16 | 0.81 | 2.4 ± 1.3 | 1.6 ± 1.2 | 29 (4) | 0.87 ± 0.55 | 0.54 | 110500 ± 51215 |
| B | 32 | 0.97 | 2.3 ± 1.3 | 1.8 ± 1.6 | 98 (113) | 1.57 ± 0.64 | 0.82 | 167797 ± 77771 |
| C | 27 | 0.93 | 1.9 ± 1.0 | 2.0 ± 1.5 | 46 (13)* | 1.30 ± 0.69 | 0.56 | 103879 ± 45021 |
| D | 3 | 0.67 | 3.2 ± 2.9 | 0.09 ± 0.01 | 4 (2)* | 0.92 ± 0.06 | 0.76 | 140979 ± 61100 |
| E | 26 | 0.96 | 3.6 ± 1.8 | 3.5 ± 2.2 | 58 (6) | 0.73 ± 0.30 | 0.34 | 63070 ± 27334 |
| F | 3 | 0.33 | 1.7 ± 1.9 | 0.71 ± 0.66 | 2 | 0.17 ± 0.30 | 0.22 | 40810 ± 17687 |
| G | 14 | 0.93 | 4.0 ± 1.3 | 3.3 ± 1.5 | 33 (6)* | 0.64 ± 0.32 | 0.40 | 74199 ± 32158 |
| H | 9 | 0.67 | 4.1 ± 1.6 | 4.1 ± 3.0 | 25 (2)* | 0.58 ± 0.47 | 0.38 | 70489 ± 30550 |
| I | 9 | 0.89 | 5.9 ± 0.6 | 3.9 ± 1.2 | 18 (2)* | 0.37 ± 0.21 | 0.24 | 44520 ± 19295 |
| J | 7 | 0.29 | 3.1 ± 1.0 | 2.1 ± 1.4 | 1 (1)* | 0.05 ± 0.13 | 0.05 | 9275 ± 4020 |
| K | 14 | 0.93 | 5.3 ± 1.4 | 2.4 ± 0.5 | 30* | 0.36 ± 0.13 | 0.26 | 48230 ± 20903 |
| L | 11 | 0.73 | 3.9 ± 0.8 | 1.9 ± 0.7 | 15* | 0.34 ± 0.25 | 0.27 | 50085 ± 21706 |
| M | 19 | 1.00 | 5.6 ± 1.5 | 2.7 ± 0.7 | 64 (18)* | 1.02 ± 1.19 | 0.52 | 96459 ± 41805 |
| N | 10 | 0.41 | 6.0 ± 1.6 | 2.1 ± 0.1 | 5 (5)* | 0.08 ± 0.12 | 0.0017 | 315 ± 137 |
| O | 15 | 0.93 | 6.7 ± 0.7 | 2.3 ± 0.3 | 35 (6)* | 0.40 ± 0.25 | 0.30 | 55650 ± 24118 |
| P | 26 | 0.58 | 5.4 ± 3.3 | 2.5 ± 2.0 | 35 (25)^+^ | 0.24 ± 0.43 | 0.29 | 53795 ± 23314 |
| Q | 16 | 0.75 | 7.9 ± 1.9 | 4.9 ± 4.1 | 33 (3) | 0.24 ± 0.18 | 0.18 | 33390 ± 14471 |
| R | 27 | 0.93 | 4.9 ± 1.0 | 3.3 ± 2.3 | 48 (4) | 0.35 ± 0.20 | 0.25 | 46375 ± 20099 |
| S | 12 | 0.92 | 4.9 ± 0.8 | 4.0 ± 1.3 | 22 (3) | 0.37 ± 0.20 | 0.24 | 44520 ± 19295 |
