# Supplementary material for: Jellyfish Support High Energy Intake of Leatherback Sea Turtles (Dermochelys coriacea): Video Evidence from Animal-Borne Cameras
Source: PLoS One. 2012 Mar 16;7(3):e33259. doi: 10.1371/journal.pone.0033259 (PMC3306388; doi:10.1371/journal.pone.0033259)
Supplement: Table S3 — Parameter estimates and significance of model terms. This table shows the linear mixed model parameter estimates and significance of model terms for three models: the effect of jellyfish encounters per dive minute on dive duration, the effect of prey size on handling time, and the effect of prey size and prey species on handling time. The results show that jellyfish encounters per dive minute are positively correlated with dive duration, that prey size is positively correlated with handling time, and that the relationship between prey size and handling time does not differ among species. The hypothesis that the residuals of these fits follow a normal distribution is not rejected by two-tailed Kolmogorov-Smirnov tests (p>0.05). (DOC) [file pone.0033259.s004.doc]

| **Behaviour** | **Effect** | **Estimate** | **Standard error** | ***T*** | ***P*** |
| --- | --- | --- | --- | --- | --- |
| Dive duration | Intercept | 0.004 | 0.0003 | 12.7 | <0.0001 |
|  | Jellyfish encounters per dive minute | 0.0001 | 0.0003 | 0.3 | 0.74 |
|  |  |  |  |  |  |
| Handling time | Intercept | 0.000014 | 0.0002 | 0.07 | 0.95 |
|  | Prey size | 0.00006 | 0.000015 | 4.04 | 0.0001 |
|  |  |  |  |  |  |
| Handling time | Intercept | 0.00002 | 0.0002 | 0.09 | 0.93 |
|  | Prey size | 0.00006 | 0.000015 | 3.94 | 0.0001 |
|  | Prey species | -0.00003 | 0.0003 | -0.10 | 0.92 |
